# Effectiveness of Household Disinfection Techniques to Remove SARS-CoV-2 from Cloth Masks

**DOI:** 10.3390/pathogens11080916

**Published:** 2022-08-15

**Authors:** Maria Angélica Monteiro Mello Mares-Guia, Anne Aline Pereira Paiva, Vinicius Motta Mello, Cristiane M. Eller, Andreza Lemos Salvio, Felipe F. Nascimento, Emanuelle S. R. F. Silva, Vinicius Tadeu Martins Guerra Campos, Ygara da Silva Mendes, Elba Regina Sampaio Lemos, Ivanildo P. Sousa, Marco Aurélio Pereira Horta

**Affiliations:** 1 Flavivírus Laboratory, Oswaldo Cruz Institute, Oswaldo Cruz Foundation, Rio de Janeiro 21040-900, Brazil; 2 Viral Hepatitis Laboratory, Oswaldo Cruz Institute, Oswaldo Cruz Foundation, Rio de Janeiro 21040-900, Brazil; 3 COVID-19 Analytical Center, Oswaldo Cruz Foundation, Rio de Janeiro 21040-900, Brazil; 4 Biosafety Level 3 Facility (BSL-3), Oswaldo Cruz Foundation, Rio de Janeiro 21040-900, Brazil; 5 Virological Technology Laboratory, Bio-Manguinhos, Oswaldo Cruz Foundation, Rio de Janeiro 21040-900, Brazil; 6 Hantavirosis and Rickettsiosis Laboratory, Oswaldo Cruz Institute, Oswaldo Cruz Foundation, Rio de Janeiro 21040-900, Brazil; 7 Enterovirus Laboratory, Oswaldo Cruz Institute, Oswaldo Cruz Foundation, Rio de Janeiro 21040-900, Brazil

**Keywords:** SARS-CoV-2, COVID-19, reuse fabric masks, disinfection

## Abstract

To assess the efficacy of washing cloth masks, we simulated SARS-CoV-2 contamination in tricoline fabric and tested decontaminants to reduce viral particles. Viral suspensions using two variants (B.1.1.28 and P.1) were inoculated in these fabrics, and the inactivation kinetics were evaluated after washing with various household disinfection products (Soap powder, Lysoform^®^, Hypochlorite sodium and 70% Alcohol), rinse numbers, and exposure times. Afterward, the fabrics were washed in sterile water, and viral RNA was extracted and amplified using RT-qPCR. Finally, viral replication in cell cultures was examined. Our findings show that all biocidal treatments successfully disinfected the tissue tested. Some products showed less reduction in viral loads, such as soap powder (1.60 × 10^4^, 1.04 × 10^3^), soap powder and Lysoform^®^ (1.60 × 10^4^, 1.04 × 10^3^), and alcohol 70% (1.02 × 10^3^, 5.91 × 10^1^), respectively. However, when sodium hypochlorite was used, this reduction was significantly increased (viral inactivation in 100% of the washes). After the first wash, the reduction in the number of viral particles was greater for the P.1 variant than for the B.1.1.28 variant (W = 51,759, *p* < 0.05). In conclusion, the role of sodium hypochlorite in cloth mask disinfection may also have implications for future health emergencies as well as recommendation by WHO.

## 1. Introduction

The ‘coronavirus disease 2019′ (COVID-19) has caused one of the biggest pandemics in the world and resulted in over 519 million confirmed cases and approximately 6.2 million deaths globally [1]. The etiological agent responsible for COVID-19 belongs to the *Coronaviridae* family [2,3]. This novel virus, named ‘severe acute respiratory syndrome coronavirus 2′ (SARS-CoV-2), consists of a glycoprotein envelope and a positive-strand RNA, and uses its envelope spike (S) protein to enter target cells [4]. Due to a proofreading mechanism that acts during viral replication, the genetic code of SARS-CoV-2 has remained remarkably stable since the first cases in China in 2019 [4,5,6]. However, many studies have revealed changes in the genome, including mutations and deletions, which are mainly associated with the S region of viral proteins. New viral variants with the potential for enhanced transmissibility have been identified as a result of these genomic changes [4,5,6,7,8].

Gamma (P.1) has emerged as a variant of concern (VOC) for global public health [6,8]. This variant originated from the formation of a subclade of the B.1.1.28 lineage. Both have circulated in Brazil since 2020. After the first epidemic peak in the city of Manaus, B.1.1.28 became the most prevalent strain from May to December 2020. It was later replaced by strain P.1 in the second epidemic peak of exponential growth, thereafter becoming the most commonly found strain in most cases in the country [7,8,9]. This Gamma VOC showed substitution mutations in its S protein, allowing it to escape neutralizing antibodies in the in vitro assays [10,11] and increasing the possibility of inter-individual transmission [12,13,14].

Since it is an airborne virus, the use of masks is a very important strategy for preventing viral transmission [15,16,17,18]. Nonetheless, the effectiveness and protection provided by masks have been debated and questioned by the media and scientific community due to the increase in the number of COVID-19 cases, the continuity of the pandemic, and the emergence of variants that have the potential for greater transmissibility [4,19]. These discussions mainly revolve around masks manufactured in homes or small family factories, which can provide protection against the virus but may be relatively less effective than surgical or N95 masks [18,20,21,22,23,24,25]. These masks can be made from different materials, such as cleaning bags, paper filters, and fabrics (linen, cotton, silk, polyester, and cotton blend) [21,22,23,24,26].

With the worsening of the COVID-19 pandemic, many countries, including Brazil, have suffered from the unavailability or high prices of surgical or N95 masks [21,27,28,29,30]. Therefore, to protect against SARS-CoV-2, people are encouraged to make their own reusable fabric masks at home at a lower cost. However, a majority of these masks will be used without quality testing by health authorities [31,32,33,34].

Concerns have been raised about the variety of cloth masks found on sale in Brazil, as well as the lack of clarity about their usefulness in blocking the virus and the efficacy of detergents and disinfectant solutions recommended by the producers for the decontamination procedure. Therefore, this study aimed to evaluate the effectiveness of different washing processes for tricoline fabric, which is commonly used to make cloth masks in Brazil, previously infected with the B.1.1.28 and P.1 variants of SARS-CoV-2.

## 2. Results

### 2.1. SARS-CoV-2 Genome Detection by RT-qPCR after Washing Processes

**Figure 1:**
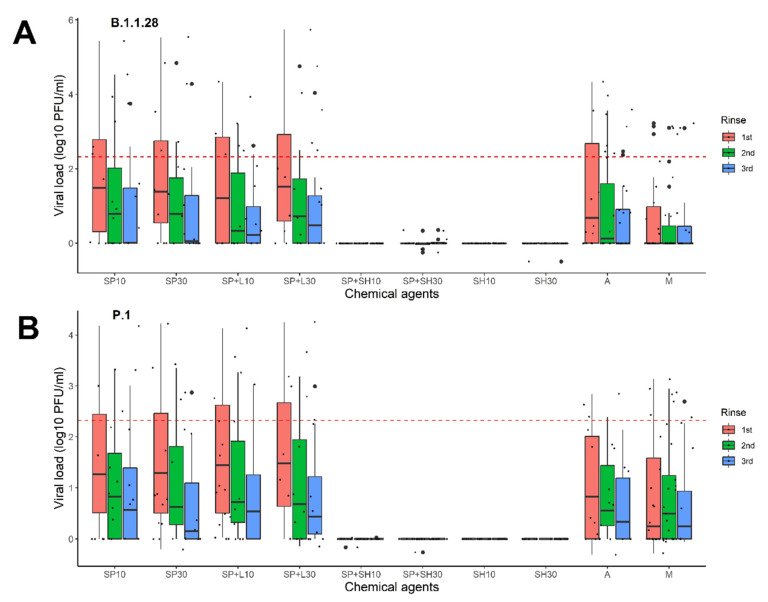
Viral loads on samples collected after different washing regimens for fabrics contaminated with SARS-CoV-2. (**A**) B.1.1.28 variant and (**B**) P.1 variant. Samples were collected after three rinses (the colors in the columns reflect the results of the viral load (VL) after the rinses, where: pink represents the VL levels after the first rinse; green represents the VL levels after the second rinse; and blue indicates the VL levels after the third rinse). SP10, soap for 10 min; SP30, soap for 30 min; SP + L10, soap and Lysoform^®^ for 10 min; SP + L30, soap and Lysoform^®^ for 30 min; SP + SH10, soap and sodium hypochlorite for 10 min; SP + SH30, soap and sodium hypochlorite for 30 min; SH10, sodium hypochlorite for 10 min; SH30, sodium hypochlorite for 30 min; A, alcohol, and M, wash mock. The dotted line is the limit of viral loads calculated from clinical samples as presented by Mello and colleagues in 2022 [18].

The viral titers were reduced by all disinfectants tested. However, the most efficient products were sodium hypochlorite and soap powder with sodium hypochlorite (Table 1). RT-qPCR confirmed that a solution containing both sodium hypochlorite and soap powder, as well as a solution containing only sodium hypochlorite, were able to remove virus particles. (Table 1, Supplementary Materials Tables S1 and S2). The statistical analyses revealed no difference between the different products depending on the soaking time (10 and 30 min) and the number of rinses performed (*p* > 0.05) (Figure 1).

### 2.2. Relationship between Viral Dilutions and Washing Products

**Figure 2:**
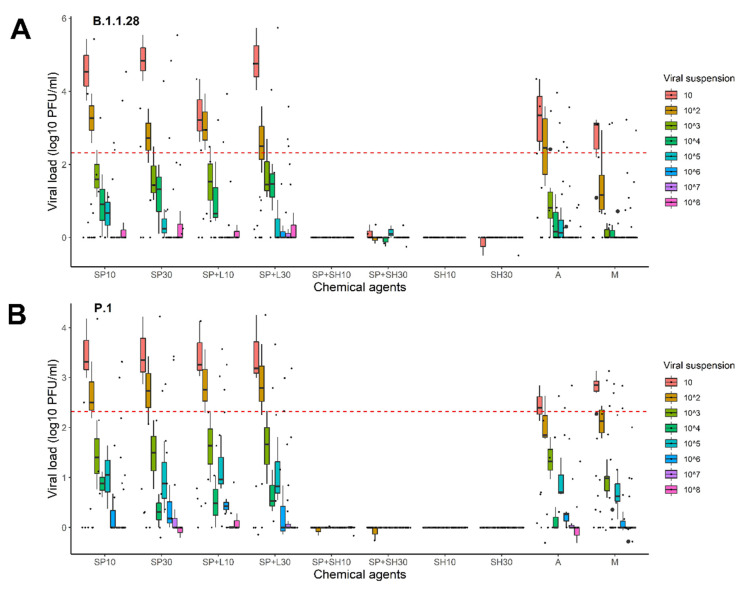
Analysis of the different virus dilutions utilized in the various processes to evaluate the effect of the disinfection agents. (**A**) B.1.1.28 variant and (**B**) P.1 variant. SP.10, soap for 10 min; SP30, soap for 30 min; SP + L10, soap and Lysoform^®^ for 10 min; SP + L30, soap and Lysoform^®^ for 30 min; SP + SH10, soap and sodium hypochlorite for 10 min; SP + SH30, soap and sodium hypochlorite for 30 min; SH10, sodium hypochlorite for 10 min; SH30, sodium hypochlorite for 30 min; A, alcohol, and M, wash mock. The dotted line is the limit of viral loads calculated from clinical samples presented by Mello and colleagues in 2022 [18].

Regardless of the soaking duration, the lower the number of virus particles in the fabric, the more efficient the disinfectant was (Figure 2). The viral load was not significantly reduced in solutions containing simply soap powder, soap powder plus Lysoform, or alcohol. As stated previously, the other washes (solutions containing only sodium hypochlorite and solutions containing soap powder and sodium hypochlorite) demonstrated full efficiency for various viral dilutions (Figure 2, Supplementary Materials Tables S1 and S2).

### 2.3. Effect of the Washing Processes on B.1.1.28 and P.1 Variants

**Figure 3:**
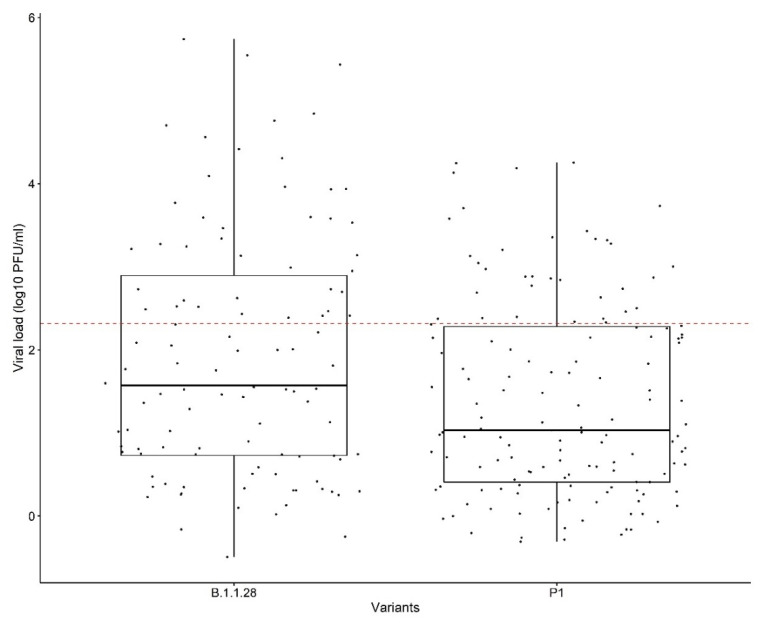
Comparison of the washing effects for the B.1.1.28 and P.1 variants. The dotted line is the limit of viral loads calculated from the clinical samples presented by Mello and colleagues in 2022 [18].

**Figure 4:**
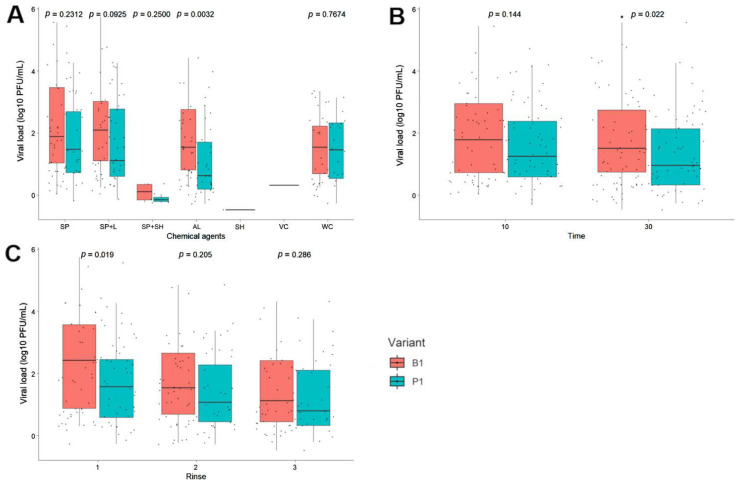
Analysis of the efficiency of the disinfectants to eliminate the B.1.1.28 and P.1 variant viral particles. (**A**) Effect of the different chemical agents, (**B**) soaking times, (**C**) and numbers of rinses in the different washes used. Commercial disinfectant products; SP, soap; SP + L, soap and Lysoform^®^; SP + SH, soap, and sodium hypochlorite; SH, sodium hypochlorite; A, alcohol; VC, virus control and M, wash mock.

When comparing the effects of the washing processes for the two SARS-CoV-2 variants used in the study, we observed that regardless of the product used, the reduction in the viral load for the P.1 variant was greater than that for the B.1.1.28 variant (W = 51,759, *p* < 0.05) (Figure 3). Additionally, the solution containing hypochlorite and soap powder was more effective at reducing viral load for both variants (B.1.1.28, *p* < 0.001; P.1, *p* < 0.001). There was a significant difference between the viral titers for the two variants at 30 min, with lower titers for the P.1 variant (*p* < 0.05). In addition, we observed that the viral load for the P.1 variant was lower than that of the B.1.1.28 (*p* < 0.05) variant after the first rinsing process (Figure 4).

### 2.4. Detecting the Presence of Virions in the Fabric Treated with Disinfectants through Cell Culturing

**Figure 5:**
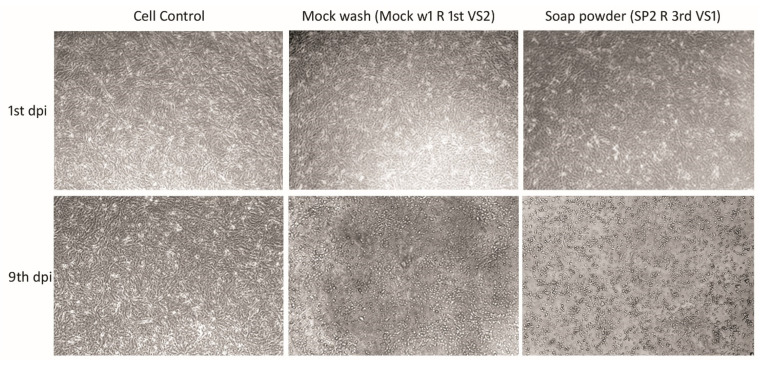
CPE on Vero CCL81 cell monolayers after control washing (Mock Wash) and washing powder disinfection. Vero CCL81 cells were inoculated with samples collected from the washing of fabrics contaminated with SARS-CoV-2. Vero cells cultures were observed on the first, fifth, seventh and ninth days after infection. Images that showed CPE were collected on the first and ninth day after infection using an Olympus IX71 microscope. The magnification is 10× for all images.

Given that RT-qPCR can detect only fragments of the genetic material and not infectious viral particles, the previously amplified samples were used to infect the Vero CCL81 cells. The infected cells were observed for nine days to assess the infectious capacity and possible presence of virions. CPE was found on the fifth day post infection for samples that had not been washed with disinfectants (mock wash), and on the ninth day post-infection for samples treated with soap or alcohol (Table 2 and Figure 5).

## 3. Discussion

Facemasks have been used to restrict viral spread during the SARS-CoV-2 pandemic [17,35]. However, because of the shortage of high-performance protective masks (e.g., N95 or FFP2), particularly in low-income countries, homemade masks, which are typically made of fabric, have been widely used as protective measures against viral spread. [35,36,37]. However, the effectiveness of such reusable masks has long been questioned [35,36,37]. Moreover, assessing the efficacy of household disinfection techniques became critical for determining whether the individual was at risk of becoming infected after reuse [36,37,38]. This study examined the efficacy of the disinfectant products that are used to wash fabric masks and evaluated whether these products decreased the load of infectious viral particles.

Different types of biocidal agents, such as alcohols and sodium hypochlorite, are used worldwide for disinfection. It has been proven that disinfectants containing 70% ethanol, or 0.1% sodium hypochlorite can reduce coronavirus contamination on surfaces within one minute of exposure [39]. 

Overall, our findings reveal that all biocidal treatments were effective at disinfection of the tested fabric. This reduction was significantly greater when sodium hypochlorite was used, and resulted in viral inactivation in 100% of the washes. According to several studies, sodium hypochlorite has high biocidal capability, particularly against respiratory viruses (including influenza and coronavirus) [40,41,42]. This efficiency could be attributed to the putative nuclease activity of the product, which achieves rapid activity against viral nucleic acids even at low concentrations. However, it is unclear whether this activity is primarily directed against the viral genome or its capsid [43].

Despite the ability of soap powder to lyse the lipidic membranes of enveloped viruses [25,44,45], such as coronaviruses, soap powder showed reduced efficacy in our study. Viral proliferation was observed in cell cultures after the use of soap alone, confirming the persistence of virions after washing. The limited efficacy could be due to the concentration utilized (as indicated by the manufacturer). However, soap powder was an effective disinfectant and caused a decrease in the virus particles in the presence of hypochlorite. It is possible that the combined activity of both the products contributed to this reduction.

Although some studies have demonstrated that 70% alcohol is effective in disinfecting SARS-CoV-2 [37,42,43], we found it to exhibit moderate efficacy. Furthermore, we detected viral growth in cell cultures after using 70% alcohol, suggesting the persistence of the infecting virus. This result could be due to the viral load used in the assay. Moreover, our findings suggest that a disinfectant can be more effective against lower virus loads. Previous studies have reported similar results, demonstrating that, in addition to frequency, concentration, and time, the amount of virus particles can alter the disinfectant’s effectiveness. [39,46]. Despite our findings, it is important to highlight that 70% alcohol and other disinfectants, such as soap powder, are still efficient at decreasing infectious loads. Therefore, they should still be used for preventative and individual hygiene, such as hand and object disinfection, as well as the washing of cloth masks. When the effects of washing were compared between the two SARS-CoV-2 variants used in the study, we found that regardless of the product used, the reduction in virus particles for the P.1 variant (W = 51,759, *p* < 0.05) was greater than that for the B.1.1.28 variant. As different viral titers were used for each variant in these experiments (2.8 × 10^8^ PFU/mL for the B.1.1.28 variant and 3.66 × 10^6^ PFU/mL for the P.1 variant), this fact may have affected the result. Furthermore, when compared to the B.1.1.28 variant, the P.1 variant revealed a higher viral particle reduction during the 30-min soak and after the first rinse step. We hypothesize that these results may be attributed to the mutations in the variants, which confer reduced resistance to disinfection agents. It is known that the P.1 variant is more contagious, more resistant to antibodies, and may show high viral loads during infection course compared with B.1.1.28 [8]. Further studies are needed to better understand these findings.

Reusable and washable fabric masks are an excellent alternative, particularly for low-income groups, when other types of masks are in limited supply. It should be noted that the number of times it is cleaned and reused may damage filtering, increasing the pore size and affect the efficacy of the mask as demonstrated by Everts et al. [41].

It’s also worth noting that our study indicates the efficacy of cloth mask cleaning techniques, which have received limited attention in the scientific literature. These findings may also have implications for the use of cloth masks during future public health emergencies. In addition, our observations support WHO recommendations [43] that the most effective and efficient disinfection approach is to clean environmental surfaces with water and detergents and then apply sodium hypochlorite.

This study has some limitations. The investigation was carried out in a laboratory, where the dilutions of each disinfectant product were prepared accurately. Furthermore, we only used one type of fabric, which is the most widely used fabric mask in Brazil (personal note). Future studies must be conducted using other fabrics.

## 4. Materials and Methods

### 4.1. Inoculation of Viral Suspensions with Tricoline Fabric

**Figure 6:**
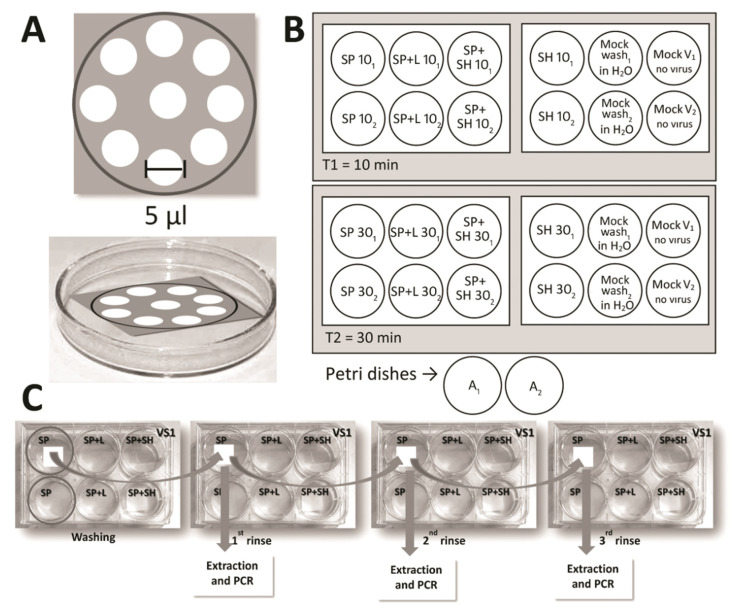
Schematic representation of the inoculations and washes. (**A**) To simulate contamination, 50 μL of the viral suspensions were distributed on the masks in 5 μL droplets. (**B**) Distribution of the different types of washes on the plates as per the viral suspension. (**C**) The rinsing and sample collection process for RNA extraction and Quantitative Reverse Transcription Polymerase Chain Reaction (RT-qPCR).

To simulate the efficacy of SARS-CoV-2 viral particle elimination in a homemade manner, we autoclaved 2 × 2 cm pieces of tricoline fabric and placed them in six-well sterile cell culture plates (CORNING^®^, Corning, NY, USA). Subsequently, a total volume of 50 µL of viral suspension was added, which was established from a standard curve in serial dilutions (10 to 10^10^) simulating different viral loads from the clinical samples. The total volume was distributed in small concentric circles of 5 µL along the cut fabric, imitating a droplet sprayer on the mask. The fabrics with viral inoculation were left for 1 h at room temperature. Figure 6 depicts the process in detail. The viral suspensions were produced from the standard strains PV010/20CoV2 (B.1.1.28) and P.1. Virus titers were determined using a 1% double-layer agarose titration assay on Vero CCL81 cell culture as previously described by Beaty et al. in 1995 [47]. The titers used in these experiments were 2.8 × 10^8^ PFU/mL for variant B.1.1.28 and 3.66 × 10^6^ PFU/mL for the variant P.1.

To guarantee the elimination of possible external contaminants, all the fabric was autoclaved before the process.

### 4.2. Washing and Soaking with Disinfectant Solutions

Washing was performed using five different commercially available disinfectant product solutions (soap powder solution, soap powder solution with Lysoform^®^, soap powder solution with sodium hypochlorite, hypochlorite solution diluted in normal water, and 70% alcohol solution) according to the manufacturer’s recommendations, as detailed in Table 3.

To evaluate the effectiveness of the washes, the virus-containing fabrics were submerged in different disinfectant solutions for 10 min or 30 min. Subsequently, they were placed on new sterile plates, washed with 2 mL plain sterile water, and shaken three times at room temperature (Figure 1B). For 70% alcohol, the fabrics sprayed with solution were evaluated 10 min or 24 h after evaporation of the product. The washing, soaking, and rinsing processes carried out in this study were used to simulate the washing process commonly used by people in Brazil (personal reference). The solutions from each rinse were collected and stored at −80 °C for subsequent molecular analysis.

Fabrics that were not treated with viral suspensions were used as negative controls (mock virus). The negative controls were also subjected to the same washing processes aforementioned. Fabrics inoculated with viral suspensions for 1 h but not treated with disinfectant products were used as positive controls and named as ‘Mock wash’. All steps of the experiment were performed in duplicate assays.

### 4.3. RNA Extraction

After soaking in disinfection products, the fabric was washed three times with sterile water. Each rinse yielded an eluate, which was then utilized as a sample for viral RNA extraction. Nucleic acids from all samples were extracted and purified using the DNA/RNA 300 kit H96 in the Janus G3 and Janus Chemagic automatic extractor (Perkin-Elmer, Waltham, MA, USA). The Janus 360 system was based on use of magnetic spheres for extracting viral nucleic acids from 300 µL of the sample. The equipment and commercial kits were used in accordance with the manufacturer’s instructions.

### 4.4. Quantitative Reverse Transcription Polymerase Chain Reaction (RT-qPCR) Detection of SARS-CoV-2 RNA

The E region of the SARS-CoV-2 genome was amplified using a molecular kit (Bio-Manguinhos, Rio de Janeiro, Brazil), as per the manufacturer’s instructions. The plate setup was automated, and analysis was performed using Janus G3 (Perkin-Elmer, Waltham, MA, USA). In this method, RT-qPCR also allowed the quantification of the viral genomic RNA of SARS-CoV-2 with the application of an in-house single-stranded RNA (ssRNA) standard curve. The commercial kit detected the E region of the genome using a FAM probe and the RP human gene using a VIC probe. Moreover, the VIC probe functions as the internal positive control for the assay. For all assays, positive and negative controls were included in the commercial kit and used in all experiments.

**Figure 7:**
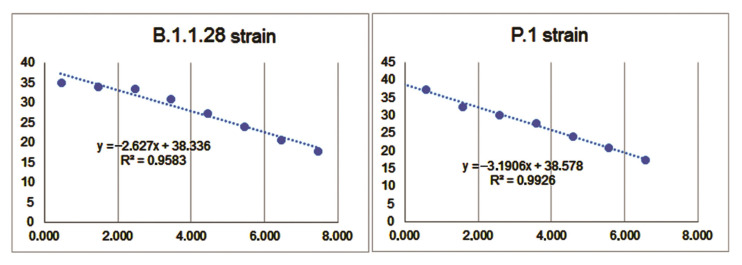
An approximate curve based on logarithmic approximation was drawn for each strain. The approximation equations and R2 values are displayed on the graph.

Samples with a cycle threshold (Ct) value lower than 38.0 for the E region were considered positive. Samples that presented Ct values greater than or equal to 40.0 were considered negative. For the RP target, a Ct value equal to or lower than 35.0 validated the experiment. Assays with a Ct value of lower than 37.0 for the positive control were validated and used for analysis. All samples with Ct values between 38.0 and 39.0 were retested. An approximate curve based on logarithmic approximation was drawn for each variant. The approximation equations and R2 values are shown (Figure 7).

### 4.5. Inoculation of Vero Cells with Virus Particles

After RT-qPCR analysis, the samples that presented with viral RNA amplification were inoculated in Vero CCL81 cells to visualize the possible cytopathic effect (CPE) and potential infectivity. To exclude any possible cytotoxic effect from the use of disinfection products, fabrics that were not treated with the viral suspension but underwent washing, soaking, and rinsing processes were used as negative controls. Fabrics that had not been washed were used as positive controls. To assess virion persistence, 100 µL of each sample was added to cells (1.2 × 10^6^ cells per well) in a 6-well flat-bottom fabric culture plate. After adsorption, the cells were incubated at 37 °C and observed on days 1, 5, 7, and 9 post infection to visualize the cytopathic effects. All tests were performed in duplicate.

### 4.6. Data Analysis

Baseline characteristics were presented as frequencies of positivity (%) and as means and medians for non-normally distributed continuous data (viral load values). The type of wash was treated as a grouping variable. Differences in the median viral load values between the washes were evaluated using the nonparametric Kruskal–Wallis test. The Mann–Whitney U test was used to compare the Cq distribution between pairs. Statistical significance was defined as a two-tailed *p*-value < 0.05. The tests were performed using the RStudio software Version 1.3.1073 (https://www.r-project.org/, accessed on 8 August 2022).

## 5. Conclusions

Cloth masks are a less expensive alternative to surgical or N95/FFP2 masks, and in the absence of medical masks, health authorities, such as the World Health Organization, support their use [48,49]. The effectiveness of household procedures in the decontamination of masks depends on the products used, viral load found on the masks, and time of contact with the decontaminant. Our findings revealed that all biocidal treatments showed different levels of effectiveness in the disinfection of the tested fabric. This reduction was significantly greater when sodium hypochlorite was used, resulting in viral inactivation in 100% of the washes. Therefore, it is plausible that the consistent use of masks can play an important role in preventing the spread of SARS-CoV-2, and these cloth masks can be reused when washed with proper products.

## Figures and Tables

**Table 1:** Data on viral load after washing with disinfection products.

	B.1.1.28 ^a^			P.1 ^b^		
	Median	Viral Load Mean	*p*-Value	Median	Viral Load Mean	*p*-Value
**Commercial disinfectant products**			<0.001			<0.001
Soap Powder	5.73	1.60 × 10^4^		5.74	1.04 × 10^3^	
Soap Powder + Lysoform^®^	4.37	1.43 × 10^4^		7.72	1.01 × 10^3^	
Soap Powder + Sodium Hypochlorite	0.0	1.44 × 10^−1^		0.0	0.04	
Sodium Hypochlorite	0.0	6.70 × 10^−3^		0.0	0.0	
70% alcohol	0.91	1.02 × 10^3^		2.56	5.91 × 10^1^	
Virus Control	0.0	4.2 × 10^−2^		0.0	0.0	
Wash Control	0.0	1.42 × 10^2^		2.51	1.14 × 10^2^	

^a^ Kruskal–Wallis = 99.425, df = 6, *p* < 0.001; ^b^ Kruskal–Wallis = 161.27, df = 6, *p* < 0.001.

**Table 2:** Analysis of viral viability in cell culture following treatment with disinfectants.

Samples Collected after Washing with Commercial Disinfectant Products	Days Post Infection							
	1st Day	Ct Value	5th Day	Ct Value	7th Day	Ct Value	9th Day	Ct Value
A.1 R 1st VS1	NO	NEG	LC	NEG	MLC	NEG	MLC	NEG
A.2 R 1st VS1	NO	NEG	LC	NEG	LC	NEG	LC	NEG
A.1 R 2nd VS1	NO	NEG	MLC	NEG	CTEcc	NEG	__	X
A.2 R 2nd VS1	NO	NEG	MLC	NEG	CTEcc	NEG	__	X
A.1 R 3rd VS1	NO	NEG	LC	NEG	MLC	NEG	MLC	NEG
A.2 R 3rd VS1	NO	NEG	LC	NEG	MLC	NEG	MLC	NEG
A.1 R 1st VS2	NO	NEG	NO	NEG	MLC	NEG	MLC	NEG
A.2 R 1st VS2	NO	NEG	NO	NEG	MLC	NEG	MLC	NEG
A.1 R 2nd VS2	NO	NEG	NO	NEG	MLC	NEG	MLC	36.87
A.2 R 2nd VS2	NO	NEG	NO	NEG	MLC	NEG	MLC	NEG
A.1 R 3rd VS2	NO	NEG	NO	NEG	LC	NEG	LC	NEG
A.2 R 3rd VS2	NO	NEG	NO	NEG	MLC	NEG	MLC	NEG
Mock w.1 R 1st VS1	NO	33.76	CPE	35.46	CPEcc	34.69	__	X
Mock w.2 R 1st VS1	NO	34.12	CPE	NEG	CPEcc	35.00	__	X
Mock w.1 R 2nd VS1	NO	35.63	CPE	38.58	CPEcc	3.57	__	X
Mock w.2 R 2nd VS1	NO	34.42	CPE	35.55	CPEcc	3.49	__	X
Mock w.1 R 3rd VS1	NO	35.06	CPE	NEG	CPEcc	36.00	__	X
Mock w.2 R 3rd VS1	NO	NEG	CPE	NEG	CPEcc	NEG	__	X
Mock w.1 R 1st VS2	NO	NEG	CPE	NEG	MLC	15.78	CPEcc	11.47
Mock w.2 R 1st VS2	NO	28.20	CPEcc	10.01	__	X	__	X
Mock w.1 R 2nd VS2	NO	NEG	CPE	NEG	MLC	NEG	MLC	NEG
Mock w.2 R 2nd VS2	NO	30.60	CPEcc	10.00	__	X	__	X
Mock w.1 R 3rd VS2	NO	__	CPE	NEG	MLC	20.43	CPEcc	13.77
Mock w.2 R 3rd VS2	NO	30.01	CPEcc	9.76	__	X	__	X
SP.1 R 3rd VS1	NO	__	LC	NEG	MLC	NEG	CPEcc	36.12
SP.2 R 3rd VS1	NO	__	NO	NEG	MLC	NEG	CEcc	27.42
SP.1 R 3rd VS2	NO	__	LC	NEG	MLC	NEG	MLC	NEG
SP.2 R 3rd VS2	NO	__	MLC	NEG	MLC	NEG	MLC	NEG
SH.1 R 3rd VS2	NO	NEG	LC	NEG	LC	NEG	LC	NEG
SH.2 R 3rd VS2	NO	35.29	LC	NEG	LC	NEG	LC	NEG
SP + L30.1 R 3rd VS1	NO	35.30	LC	NEG	LC	NEG	LC	NEG
SP + L30.2 R 3rd VS1	NO	NEG	MLC	NEG	MLC	NEG	MLC	NEG
SP + L30.1 R 3rd VS2	NO	NEG	MLC	NEG	MLC	NEG	MLC	NEG
SP + L30.2 R 3rd VS2	NO	NEG	MLC	NEG	MLC	NEG	MLC	NEG

NO, no effect; CPE, cytopathic effect; CPEcc, cytopathic effect and collected cells; CTEcc, cytotoxic effect and collected cells; LC, loose cells; MLC, many loose cells; NEG, negative; POS, positive. Commercial disinfectant products; SP, soap; SP + L, soap and Lysoform^®^; SP + SH, soap, and sodium hypochlorite; SH, sodium hypochlorite; A, alcohol; and MW, wash mock.

**Table 3:** Dilution of disinfectant products according to the manufacturer’s recommendations.

Solution	Volume	Volume of Water
Soap powder	1/8 cup (or 11.20 g) of soap powder ^b^	13.5 L
Soap powder + Lysoform^®^	1/8 cup (or 11.20 g) of soap powder + 25 mL of Lysoform^®^	13.5 L
Soap powder + Hypochlorite sodium	1/8 cup (or 11.20 g) of soap powder + 10 mL of Hypochlorite	13.5 L
Hypochlorite sodium	10 mL of Hypochlorite	0.5 L
70% alcohol	300 mL of 92.8% ^a^	0.7 L

^a^ Alcohol commonly sold in Brazilian markets; ^b^ according to the manufacturer, the components in the soap powder used in this experiment are: anionic surfactants, buffers, dye, optical brightener, sodium alkyl benzene sulfonate, and fragrance.

## Data Availability

Not applicable.

## Supplementary Materials

The following supporting information can be downloaded at: https://www.mdpi.com/article/10.3390/pathogens11080916/s1. Table S1: Overall wash results for the B1.1.28 variant; Table S2: Overall wash results for the P.1 variant.
