# Supplementary material for: Effectiveness of Household Disinfection Techniques to Remove SARS-CoV-2 from Cloth Masks
Source: Pathogens. 2022 Aug 15;11(8):916. doi: 10.3390/pathogens11080916 (PMC9415727; doi:10.3390/pathogens11080916)
Supplement: Supplementary file 1 [file pathogens-11-00916-s001.zip › pathogens-1829541-supplementary.pdf]

Effectiveness of household disinfection techniques to remove SARS-CoV-2 from cloth masks

Supplementary Materials

Supplementary Table S1

Chemical Agents	viral suspension (Log 10)	B.1.1.28											
		Soak Time											
		10 min						30 min					
		1st Rinse[a]		2nd Rinse[a]		3rd Rinse[a]		1st Rinse[a]		2nd Rinse[a]		3rd Rinse[a]	
		Ct value mean	Viral Load mean[b]	Ct value mean	Viral Load mean[b]	Ct value mean	Viral Load mean[b]	Ct value mean	Viral Load mean[b]	Ct value mean	Viral Load mean[b]	Ct value mean	Viral Load mean[b]
Soap Powder	Log^{1}	24,1	2,72 x 10^{5}	26,45	3,65 x 10^{4}	28,51	5,88 x10^{3}	23,8	3,52 x 10^{5}	25,61	7 x 10^{4}	27,09	2.03 x 10^{4}
	Log^{2}	28	8,58 x 10^{3}	29,74	1,88 x 10^{3}	31,52	3,94 x 10^{2}	29,06	3,4 x10^{3}	31,18	5,37 x 10^{2}	32,95	1,13 x 10^{2}
	Log^{3}	32,03	2,58 x 10^{2}	34,14	3,96 x 10^{1}	35,42	1,30 x 10^{1}	31,79	3,3 x 10^{2}	34,57	2,71 x 10^{1}	35,65	1,09 x 10^{1}
	Log^{4}	33,81	5,68 x 10^{1}	35,93	1,04 x 10^{1}	38,28	1,05	33,11	9,76 x 10^{1}	34,87	2,39 x 10^{1}	38,07	1,35
	Log^{5}	35,04	1,94 x 10^{1}	36,57	5,55	40	0	36,31	5,92	37,69	1,87	40	0
	Log^{6}	40	0	40	0	40	0	40	0	40	0	40	0
	Log^{7}	40	0	40	0	40	0	40	0	40	0	40	0
	Log^{8}	37,26	2,61	40	0	40	0	36,43	5,32	40	0	40	0
Soap Powder + Lysoform	Log^{1}	26,94	5,04 x 10^{4}	29,89	1,64 x 10^{3}	31,46	5,38 x 10^{2}	23,25	5,51 x 10^{5}	26	5,74 x 10^{4}	27,72	1,24 x 10^{4}
	Log^{2}	28	8.66 x 10^{3}	30,59	8,92 x 10^{2}	32,06	2,44 x 10^{2}	28,91	3,82 x 10^{3}	31,76	3,34 x 10^{2}	33,67	6,9 x 10^{1}
	Log^{3}	31,8	3,08 x 10^{2}	34,31	3,41 x 10^{1}	37	3,23	31,38	4,98 x 10^{2}	34,51	3,34 x 10^{1}	35,42	1,35 x 10^{1}
	Log^{4}	32,88	1,44 x 10^{2}	36,62	6,47 x 10^{1}	37,15	3,2	33,05	1,02 x 10^{2}	34,48	2,95 x 10^{1}	36,39	5,51
	Log^{5}	40	0	40	0	40	0	35,64	1,06 x 10^{1}	40	0	40	0
	Log^{6}	40	0	40	0	40	0	37,48	2,12	40	0	40	0
	Log^{7}	40	0	40	0	40	0	40	0	40	0	37,74	1,69
	Log^{8}	40	0	40	0	40	0	36,54	5,62	40	0	40	0
Soap Powder + Sodium Hypochlorite	Log^{1}	40	0	40	0	40	0	40	0	40	0	37,41	2,25
	Log^{2}	40	0	40	0	40	0	40	0	38,76	6,90 x 10^{-1}	40	0
	Log^{3}	40	0	40	0	40	0	40	0	40	0	40	0
	Log^{4}	40	0	40	0	40	0	40	0	38,99	5,64 x 10^{-1}	40	0
	Log^{5}	40	0	40	0	40	0	40	0	37,46	2,16	38,07	1,26
	Log^{6}	40	0	40	0	40	0	40	0	40	0	40	0
	Log^{7}	40	0	40	0	40	0	40	0	40	0	40	0
	Log^{8}	40	0	40	0	40	0	40	0	40	0	40	0
Sodium Hypochlorite	Log^{1}	40	0	40	0	40	0	40	0	40	0	39,63	3,22 x 10^{-1}
	Log^{2}	40	0	40	0	40	0	40	0	40	0	40	0
	Log^{3}	40	0	40	0	40	0	40	0	40	0	40	0
	Log^{4}	40	0	40	0	40	0	40	0	40	0	40	0
	Log^{5}	40	0	40	0	40	0	40	0	40	0	40	0
	Log^{6}	40	0	40	0	40	0	40	0	40	0	40	0
	Log^{7}	40	0	40	0	40	0	40	0	40	0	40	0
	Log^{8}	40	0	40	0	40	0	40	0	40	0	40	0
70% alcohol[c]	Log^{1}	27,92	9,19 x 10^{3}	30,1	1,36 x 10^{3}	31,85	2,93 x 10^{2}	26,94	2,61 x 10^{4}	28,98	3,97 x 10^{3}	32,11	2,58 x 10^{2}
	Log^{2}	28,9	3,92 x 10^{3}	32,29	2,02 x 10^{2}	34,3	3,56 x 10^{1}	29,24	2,92 x 10^{3}	31,45	4,21 x 10^{2}	34,64	2,9 x 10^{1}
	Log^{3}	35,99	7,93	37,12	2,98	36,39	5,5	31,99	2,71 x 10^{2}	34,75	2,31 x 10^{1}	40	0
	Log^{4}	40	0	40	0	40	0	35,21	3,16 x 10^{1}	36,19	6,73	37,52	2,04
	Log^{5}	36,9	3,87	37,65	1,82	40	0	36,18	6,93	40	0	40	0
	Log^{6}	37,55	1,99	40	0	40	0	40	0	40	0	40	0
	Log^{7}	40	0	40	0	40	0	40	0	40	0	40	0
	Log^{8}	40	0	40	0	40	0	40	0	40	0	40	0
Wash Control	Log^{1}	30,09	1,38 x 10^{3}	32,94	1,22 x 10^{2}	35,48	1 x 10^{2}	29,86	1,76 x 10^{3}	30,19	1,63 x 10^{2}	30,21	2,19 x 10^{3}
	Log^{2}	33,69	5,88 x 10^{1}	36,21	6,55	36,62	4,82	30,63	9,8 x 10^{2}	34,34	3,35 x 10^{1}	36,37	6,42
	Log^{3}	37,57	1,96	40	0	37,32	2,44	40	0	40	0	40	0
	Log^{4}	40	0	37,67	1,79	40	0	40	0	37,42	2,23	40	0
	Log^{5}	36,45	5,22	40	0	40	0	40	0	40	0	40	0
	Log^{6}	40	0	40	0	40	0	40	0	40	0	40	0
	Log^{7}	40	0	40	0	40	0	40	0	40	0	40	0
	Log^{8}	40	0	40	0	40	0	40	0	40	0	40	0
Virus Control	Log^{1}	40	0	40	0	40	0	40	0	40	0	40	0
	Log^{2}	40	0	40	0	40	0	40	0	37,52	2,04	40	0
	Log^{3}	40	0	40	0	40	0	40	0	40	0	40	0
	Log^{4}	40	0	40	0	40	0	40	0	40	0	40	0
	Log^{5}	40	0	40	0	40	0	40	0	40	0	40	0
	Log^{6}	40	0	40	0	40	0	40	0	40	0	40	0

	**Log^{7}**	40	0	40	0	40	0	40	0	40	0	40	0
	**Log^{8}**	40	0	40	0	40	0	40	0	40	0	40	0

a = All experiments were performed in duplicates, the data presented in the table are the means of Cts and viral loads from rinses; b = Viral loads are presented in the concentration of PFU/ml.

Supplementary Table S2

**P1**													
		**Soak Time**											
		**10 min**						**30 min**					
**Chemical Agents**	**Viral Suspension (Log^{10})**	**1st Rinse^{a}**		**2nd Rinse^{a}**		**3rd Rinse^{a}**		**1st Rinse^{a}**		**2nd Rinse^{a}**		**3rd Rinse^{a}**	
		**Ct value mean**	**Viral Load mean^{b}**	**Ct value mean**	**Viral Load mean^{b}**	**Ct value mean**	**Viral Load mean^{b}**	**Ct value mean**	**Viral Load mean^{b}**	**Ct value mean**	**Viral Load mean^{b}**	**Ct value mean**	**Viral Load mean^{b}**
**Soap Powder**	**Log^{1}**	25,24	1,54 x 10^{4}	28	2,1 x 10^{3}	29	5,4 x 10^{3}	25,1	1,77 x 10^{4}	27,87	2.27 x 10^{3}	29,42	7,68 x 10^{2}
	**Log^{2}**	27,98	2,17 x 10^{3}	30,59	3,18 x 10^{2}	31,6	1,53 x 10^{2}	27,64	2,7 x 10^{3}	29,85	5,44 x 10^{2}	31,98	1,22 x 10^{2}
	**Log^{3}**	31,73	1,41 x 10^{2}	34,11	2,52 x 10^{1}	36,1	5,57	31,75	1,41 x 10^{2}	33,78	3,25 x 10^{1}	36,11	5,92
	**Log^{4}**	35,75	7,7	36,65	4,02	34,8	1,34 x 10^{1}	36,44	4,67	37,59	2,04	40	0
	**Log^{5}**	33,35	4,46 x 10^{1}	35,22	1,12 x 10^{1}	37,36	2,36	33,05	5,38 x 10^{1}	35,77	7,91	37,64	1,97
	**Log^{6}**	36,41	5,08	40	0	40	0	35,85	7,12	38,01	1,51	40	0
	**Log^{7}**	40	0	40	0	40	0	37,42	2,31	40	0	40	6,25x 10^{-1}
	**Log^{8}**	40	0	40	0	40	0	39,23	0	40	0	40	0
**Soap Powder + Lysoform**	**Log^{1}**	25,38	1,36 x 10^{4}	28,16	1,9 x 10^{3}	28,91	1,11 x 10^{3}	25	1,8 x 10^{4}	28,42	1,6 x 10^{3}	29,03	1,01 x 10^{3}
	**Log^{2}**	27,19	3,8 x 10^{3}	29,78	5,92 x 10^{2}	31,24	2,03 x 10^{2}	26,87	5,09 x 10^{3}	29,66	7,24 x 10^{2}	31,39	1,82 x 10^{2}
	**Log^{3}**	31,22	2,38 x 10^{2}	33,36	3,26 x 10^{1}	35,68	1,02 x 10^{1}	31,14	2,15 x 10^{2}	33,28	4,58 x 10^{1}	35,78	8,86
	**Log^{4}**	35,25	1,27 x 10^{1}	37,02	3,22	0	0	34,88	1,45 x 10^{1}	36,88	3,89	37,54	2,12
	**Log^{5}**	32,69	7,27 x 10^{1}	35,48	9,2	36,07	6,2	32,81	6,59	35,93	1,02 x 10^{1}	36,83	3,52
	**Log^{6}**	36,73	4,44	37,18	2,89	37,66	1,94	35,88	8,98	39,04	7,16 x 10^{-1}	40	0
	**Log^{7}**	38,5	1,06	40	0	40	0	40	0	40	0	38,18	1,33
	**Log^{8}**	37,7	1,88	40	0	40	0	40	0	40	0	40	0
**Soap Powder + Sodium Hypochlorite**	**Log^{1}**	40	0	40	0	40	0	40	0	40	0	40	0
	**Log^{2}**	39,1	6,86 x 10^{-1}	40	0	40	0	40	0	39,41	5,49 x 10^{-1}	40	0
	**Log^{3}**	40	0	40	0	40	0	40	0	40	0	40	0
	**Log^{4}**	40	0	40	0	40	0	40	0	40	0	40	0
	**Log^{5}**	40	0	40	0	40	0	40	0	40	0	40	0
	**Log^{6}**	40	0	40	0	40	0	40	0	40	0	40	0
	**Log^{7}**	40	0	40	0	40	0	40	0	40	0	40	0
	**Log^{8}**	40	0	40	0	38,5	1,06	40	0	40	0	40	0
**Sodium Hypochlorite**	**Log^{1}**	40	0	40	0	40	0	40	0	40	0	40	0
	**Log^{2}**	40	0	40	0	40	0	40	0	40	0	40	0
	**Log^{3}**	40	0	40	0	40	0	40	0	40	0	40	0
	**Log^{4}**	40	0	40	0	40	0	40	0	40	0	40	0
	**Log^{5}**	40	0	40	0	40	0	40	0	40	0	40	0
	**Log^{6}**	40	0	40	0	40	0	40	0	40	0	40	0
	**Log^{7}**	40	0	40	0	40	0	40	0	40	0	40	0
	**Log^{8}**	40	0	40	0	40	0	40	0	40	0	40	0
**70% alcohol^{c}**	**Log^{1}**	29,5	7,42 x 10^{2}	30,94	2,5 x 10^{2}	31,76	1,37 x 10^{2}	28,5	1,46	31,9	1,27 x 10^{2}	34,26	2,25 x 10^{1}
	**Log^{2}**	30,18	4,28 x 10^{2}	32,92	5,92 x 10^{1}	32,68	7,22 x 10^{1}	29,65	6,96 x 10^{2}	33,29	4,75 x 10^{1}	35,26	1,08 x 10^{1}
	**Log^{3}**	32,82	6,82 10^{1}	34,35	2,15 x 10^{1}	35,47	9,43	33,08	5,30 x 10^{1}	33,73	3,57 x 10^{1}	36,89	3,38
	**Log^{4}**	40	0	37,27	2,57	0	0	38,1	3,45	38,8	8,52 x 10^{-1}	39,29	5,98 x 10^{-1}
	**Log^{5}**	34,11	2,56	36,32	5,1	36,45	5,97	37,26	3,89	38,49	1,07	38,87	8,1
	**Log^{6}**	37,74	1,83	37,58	2,05	40	0	37,4	4,18	37,47	2,22	38,08	1,56
	**Log^{7}**	40	0	38,3	1,22	40	0	40	0	40	0	38,12	1,39
	**Log^{8}**	40	0	39,56	4,92 x 10^{-1}	40	0	38,62	1,22	39,09	6,91 x 10^{-1}	38,58	9,99 x 10^{-1}
**Wash Control**	**Log^{1}**	29,53	9,42 x 10^{2}	29,41	7,62 x 10^{2}	31,32	1,95 x 102	28,58	1,35 x 10^{3}	29,18	1,44 x 10^{2}	29,99	4,9 x 10^{2}
	**Log^{2}**	30,97	2,42 x 10^{2}	32,64	2,89 x 102	32,9	1,4 x 102	31,4	1,86 x 10^{2}	30,82	2,37 x 10^{2}	32,18	1,01 x10^{2}
	**Log^{3}**	35,4	2,19 x 10^{2}	35,45	9,56	37,21	2,75	34,23	2,44 x 101	35,27	1,53 x 10^{1}	36,68	3,93
	**Log^{4}**	37,44	2,27	40	0	40	0	40	0	40	0	40	0
	**Log^{5}**	38,05	9,19 x 10^{1}	36,55	4,31	36,6	4,71	34,88	3,05 x 10^{1}	35,53	1,16 x 10^{1}	37	3,15
	**Log^{6}**	38,04	1,47	40	0	40	0	37,57	2,07	40	0	38,75	8,83x10^{-1}
	**Log^{7}**	40	0	40	0	40	0	39,47	5,25x10-1	40	0	38,68	9,29x10^{-1}
	**Log^{8}**	40	0	40	0	40	0	40	0	40	0	40	0
**Virus Control**	**Log^{1}**	40	0	40	0	40	0	40	0	40	0	40	0
	**Log^{2}**	40	0	40	0	40	0	40	0	40	0	40	0
	**Log^{3}**	40	0	40	0	40	0	40	0	40	0	40	0

	**Log[4]**	40	0	40	0	40	0	40	0	40	0	40	0
	**Log[5]**	40	0	40	0	40	0	40	0	40	0	40	0
	**Log[6]**	40	0	40	0	40	0	40	0	40	0	40	0
	**Log[7]**	40	0	40	0	40	0	40	0	40	0	40	0
	**Log[8]**	40	0	40	0	40	0	40	0	40	0	40	0

a = All experiments were performed in duplicates, the data presented in the table are the means of Cts and viral loads from rinses; b = Viral loads are presented in the concentration of PFU/ml.
